# Ten Simple Rules for Finishing Your PhD

**DOI:** 10.1371/journal.pcbi.1003954

**Published:** 2014-12-04

**Authors:** Jacopo Marino, Melanie I. Stefan, Sarah Blackford

**Affiliations:** 1 Department of Chemistry, University of Zurich, Zurich, Switzerland; 2 Department of Neurobiology, Harvard Medical School, Boston, Massachusetts, United States of America; 3 Society for Experimental Biology (SEB), Lancaster University, Lancaster, United Kingdom; National Institutes of Health, United States of America

## Introduction

After years of research and with completion in sight, the final year of the PhD often represents the most challenging time of a student's career, in which the ultimate reward is the PhD honor itself. A large investment in time, energy, and motivation is needed, with many tasks to be completed; concluding experiments must be carried out, results interpreted, and a research story mapped out in preparation for writing the final thesis. All the while, administrative obligations need attention (e.g., university credits and mandatory documents), papers may need to be published, students mentored, and due consideration paid to planning for the next career move. Without some form of strategic action plan and the employment of project management skills, students run the risk of becoming overwhelmed and run down or of not meeting their final deadlines. Personal time management and stress resilience are competences that can be developed and honed during this final period of the PhD.

Here, we present ten simple rules on how to deal with time issues and conflict situations when facing the last year of a PhD in science. The rules focus on defining research goals in advance and designing a plan of action. Moreover, we discuss the importance of managing relationships with supervisors and colleagues, as well as early career planning.

## Rule 1: Plan Your Last Year in Advance

Preparing a plan of action for the final year of your PhD is vital. Ideally, devised and agreed upon with your supervisor, a plan will help to optimize the time left and reduce feelings of being overwhelmed. Individuals plan in different ways; some prefer to work towards their goals in a stepwise linear fashion, whilst others are more comfortable flitting from task to task until all the jobs are done. There is no definitive way to plan, so find out what works best for you. You may decide to map out a timeline, or perhaps a mind-map is your preferred planning style. Whichever method you use, it's important that you adhere to your plan whilst allowing for some flexibility (but not distraction or procrastination).

Your time frame will vary according to the organization of your graduate school, your supervisor or advisory committee, and even your graduation date, but one year before submission of your doctoral thesis is the time when you should decide on how best to invest the last months of your research and associated activities. Having a plan of action will help to avoid time wasting, e.g., being distracted by superfluous experiments that might be interesting but are not necessary. Furthermore, from a psychological point of view, referring to a concrete plan can make you feel more secure and in control. Ideally, the supervisor and PhD student should both agree on the overall plan (with provision for the unexpected, e.g., technical issues), with intermittent reviews every few weeks to check that progress is being made. Your supervisor should also be able to advise you on the organization and writing of your thesis—for example, its structure—and the number and length of chapters to include.

## Rule 2: Make Your Priorities Clear

Select the activities you want to include in your plan. What are your priorities? They are likely to include experiments that will give the thesis a conclusion or that may be necessary to publish a final paper. Mandatory administrative tasks will also need attention, and allowing time to prepare for your next career move will give you the best chance of a seamless and successful transition post-PhD. As a final year PhD candidate, you are likely to have acquired high-level competencies comparable to those of a junior postdoctoral researcher, in which case your supervisor may offer you responsibility for new projects or graduate students. Saying no to him/her can be difficult for various reasons, e.g., fear of potentially creating conflict in your relationship or causing a negative reaction or of perhaps losing the opportunity to be included in future research activities and publications. It can also be difficult to let go of a topic or project to which you are wedded or to miss out on the opportunity to help train the next generation of scientists. In such situations, referring back to your plan (Rule 1), previously agreed upon with your supervisor, should help to remind you both of your priorities and deadlines, making negotiation easier. However, should any conflict of opinion arise between you, bear in mind that finding a mutually agreeable solution is the best way forward. You can take advice from a mentor or refer to the many publications that provide approaches and tactics for effective negotiation. If the relationship between you and your supervisor is more complicated and cannot be resolved by a discussion, you may need to turn to your graduate school, your academic committee, or other senior managers in your institution, who can act to mediate the situation.

## Rule 3: “The Truth Can Wait”

A research project is never really finished, so do not try to do everything before submitting. In fact, the perfect doctoral thesis does not exist; there are students with good research projects and many publications and others with more difficult and testing challenges who are still waiting for their first paper. If the project is ambitious, it might take several years to reach the final goal, and thus the thesis might only be a small part of the whole story. If the project is going well, it will open up new research questions and future directions, some of which will be beyond the scope of a PhD. At some point, you need to decide that what you have is enough for a PhD and start writing (a strategy we heard described at a dissertation-writing seminar in Cambridge as “the truth can wait”; it helps to write this on a post-it note and stick it on your computer!). Starting to write the thesis is not easy when there is a sense that more could be done to accumulate more data and a fuller story; a common mistake is to go back to the lab instead of getting started with the results chapters of the thesis. To postpone writing will cause delays and not necessarily improve the thesis whilst increasing the prospect of unfulfilled and extended deadlines. Thus, once the experiments that you have agreed on have been completed, it is really important to start writing with the data in hand.

## Rule 4: Enlist Support

Finalizing experiments and writing the thesis (and even papers), as well as considering your next career transition, can be stressful and even isolating. It is a contrast to the relatively more relaxed earlier years of the PhD experience, and the writing process does not come naturally to everyone. The prospect of facing these stresses alone can make the experience even harder to bear, so it is advisable to communicate with and find support in those you trust and respect. Relying on such people during this period can help to ease the strain and enable you to achieve your final aims so that you arrive at your PhD graduation with your sanity still intact! Talking about personal feelings with selected colleagues usually helps you to realize that you are not alone, whatever difficulties and challenges you might be experiencing with your research project, supervisor, or coworkers. Sharing uncertainties and talking through issues can be constructive, helping you to understand the strategies other people use to cope with similar problems. As well as colleagues, it can also help to talk to friends and family, even though they won't be as au fait with the highly particular challenges you are experiencing. You can share your feelings and anxieties with them, but they can also act as a welcome distraction to help you to relax and take a break from thinking about the stresses of your PhD.

Support and advice can also come in the shape of courses, books, blogs, mentoring, etc. There is much published on the subject of how to write a thesis [1]. Furthermore, graduate schools, such as those in which we are based, usually offer courses to help PhD candidates improve their personal and professional skills. For example, the University of Zurich organizes courses on, amongst others, time and self-management skills, managing conflict, and academic writing and publishing [2]. The Graduate School of Arts and Sciences at Harvard lists workshops and resources offered across the university on topics such as scientific writing, time management, and overcoming procrastination. In addition to relying on your supervisor, postdoctoral researchers in your group or department (or even friendly collaborators) may agree to read chapters of your thesis and comment on aspects such as content, logical flow of ideas, and the overall structure. At a later stage, you may want to engage someone to check your grammar, spelling, and reference style (this can be especially important if you are not writing in your native language). If your PhD defense includes a presentation, try to practice beforehand, preferably in front of some of your peers, and include asking for feedback and possible questions that may come up. This should make you feel more prepared and confident.

## Rule 5: Get Familiar with the Software

Being familiar with software for both writing and making figures will facilitate the creation of your thesis. One of the most effective tools with which to produce a scientific document is LaTeX (www.latex-project.org). This software, freely available, is not as immediately understandable as other text editors, but the advantages are greater: it offers a professional layout similar to published books, it makes the insertion and management of figures easier as their position in the file does not depend on text editing, and it allows for easy typesetting of mathematical equations and referencing of articles from a bibliography database. Moreover, the text file size does not increase while inserting figures, making its handling easier. An example LaTeX package for typesetting dissertations is “classicthesis”, written by André Miede (http://www.miede.de/index.php?page=classicthesis). Although advantageous, LaTeX can also present disadvantages. In contrast to commonly used text editors (e.g., Microsoft Word), it does not make it easy to track changes in the manuscript, often a preferred way for supervisors to correct theses in an electronic form. Therefore, we suggest you discuss the preferred software with your supervisor when you agree upon your plan (Rule 1).

A professional design software can also speed up the creation of figures for your thesis, which can be further used for your final PhD presentation, so check whether your institution provides an introductory course to some of these software packages. Taking a one-day class can save you a lot of time later. Organize your bibliography; many excellent reference managers exist that allow you to catalogue and annotate the papers you have read and integrate them seamlessly with text processing software (e.g., Endnote or the freely available Mendeley and Readcube). Choose one that fits your needs and check whether your university provides institutional licenses (and be disciplined about adding each paper you read to it!).

Consider using version control software. This allows you to keep a log of all the changes you make to a file or directory and makes it easy to recover a previous version if something goes wrong or to merge two versions of a file. This is often used in software projects to produce, document, and improve computer code, but it can also be useful when working on a long text document, such as a dissertation. Commonly used free version control systems include git/GitHub [git, github], Subversion [svn], and Bazaar [bzr] (see Table 1).

**Table 1:** List of free version control systems.

Version Control System	Developer	Available:
[jabref]	JabRef Development Team (2014) JabRef [Software]	http://jabref.sf.net
[git]	Git Development Team (2014) Git [Software]	http://git-scm.com/
[github]	GitHub Development Team (2014) GitHub [Software]	https://github.com/
[svn]	Apache Software Foundation (2014) Subversion [Software]	https://subversion.apache.org/
[bzr]	GNU Project (2014) Bazaar [Software]	http://bazaar.canonical.com/en/

Most important of all is to have a backup strategy. A hard-drive crash at the wrong moment can set your work back by weeks and jeopardize the timely completion of your thesis. Institutions or departments will often have a backup system employees can make use of. This may require you to install a specific piece of software on your computer that backs up your data at regular intervals or to save your file on an institute server. Contact the information technology (IT) department at your institute to learn about your options.

## Rule 6: Know Your University's Procedures and Regulations

During the course of your PhD, you will have been acquiring project management skills, such as organizing your time and resources, reviewing progress, and meeting deadlines. In order to avoid last-minute surprises, you can capitalize on and develop these skills during the final year of your PhD. Prepare a list of all the documents and certificates that you will need, even before you start writing; it will be of critical importance to include this information in your plan and priorities (Rules 1 and 2). Having a good working relationship with someone who can help you to navigate a bureaucratic process will usually be an asset and will ensure you are familiar and aware of all the rules. Considering the amount of documents and certificates that are needed for handing in a thesis, it is advantageous to introduce yourself to the institute secretary or human resources manager, as well as any other staff who can help you to deal with the administrative side of the process. Don't rely on previous documents, which may have been revised since the last person in your group graduated. Be aware of all the necessary institutional administrative requirements (e.g., credit points, research seminar attendance, publications, etc.), as well as the faculty criteria, including deadlines (as well the date of the graduation ceremony), thesis copy numbers and format, font size, binding, and supporting documents. Take time to go through the list of documents and start collecting them in a folder. Get the formatting right early on, e.g., by using a dedicated template file. With your documents in order, you are bound to feel you have the situation more under control, which can help to reduce stress and enable you to focus more closely on writing your thesis.

## Rule 7: Exploit Synergies

You are doing a lot of work for your thesis, so use it to your advantage. The literature review in your introduction can also be used to write and publish a future review article, an idea that might also be welcomed by your supervisor. If you are intending to write a grant proposal for a postdoctoral fellowship on a similar research topic, you can use some of the thesis introduction and future directions as a basis for your research plan. If you are keen to gain teaching experience, you could propose a short course on your specialty area. For instance, at Harvard Medical School, senior graduate students and postdoctoral researchers can be involved in lecturing on short, specialized “nanocourses” [3]. You may also be able to deliver a specialized lecture within a class your supervisor is teaching or, ideally after you have completed the PhD, teach at a workshop or summer school.

Take advantage of opportunities to deliver a talk as an invited speaker at a conference or at another institute, for example, if you are visiting a research group or investigating possible postdoctoral options. This will give you the chance to practice your defense presentation in front of an unfamiliar audience and, at the same time, allow a potential future supervisor and colleagues to gain a more complete picture of your research interests, skills, and personality.

## Rule 8: Pay Attention to Your Career

It is not always easy to decide on which career path to follow after your PhD. You have been trained primarily towards an academic research career, and so many PhD graduates choose to continue on with a postdoctoral position as their first career destination. This is perfectly acceptable, and many industrial employers look upon early-career postdoctorals favorably. However, it is worth bearing in mind that permanent tenured positions are hard to secure nowadays and competition is tough, with less than 5% of those who complete a PhD ultimately realizing an academic career [4]. For those who are determined to have an academic career, a strategic research plan is crucial; for those who are unsure, a viable alternative career plan is equally important.

Knowledge of your professional and personal skills and capabilities, personality, values, and interests, as well as how to map them onto the job market and sell them to employers, will help you to make effective career decisions and a successful transition to your next job. In addition, factors such as your personal situation and priorities, mobility, and preferred work–life balance all need to be taken into consideration before entering the complicated world of the job market. Be ready to make compromises either in your work or personal life, depending on your priorities. Take advantage of courses and professional career guidance and coaching while you are still at university, as they are usually offered free of charge. Along with books and websites, face-to-face career support can help raise your self-awareness and knowledge of the job market so you can start to decide which types of career may best suit you. Blackford's book and blog [4] contain useful material on career planning for bioscientists, with concrete examples of different career paths within and outside of academia, and further information and resources. In addition, the *Science*Careers portal offers an online tool [5] to create an individual development plan and explore your career options based on your skills, interests, and values. Also, take advantage of dedicated career job boards associated with specialist websites, such as that of the International Society for Computational Biology [6].

How soon should you start job seeking? Finding a job whilst writing up your thesis can seem like an attractive prospect, but it's important to consider that applying for jobs can easily take up as much time as working a full-time job. Then, if you do secure a job, the time left for writing up your thesis, completing experiments, and wrapping up your lab work will be seriously limited. It is exceedingly hard to write a doctoral thesis in the evenings after work or on the weekends, so in case you are offered a job before you have finished the PhD, consider seriously how this might affect your work and life. On the other hand, finishing a PhD when scholarship money has been seriously reduced (or has run out) comes with a different set of challenges. Many students need to tap into their savings (if indeed they have any), drastically reduce their spending, and move out of their accommodation. Losing employment at the university can also affect health insurance, social security, and visa status. Finishing up a PhD under these additional constraints and pressures can be extremely challenging, both logistically and psychologically. To ensure that you can concentrate all your time on (and get paid for) finishing your PhD, start planning ahead one year earlier. Be aware of your university's regulations, talk to your supervisor about the funding situation (is it possible for you stay on as a postdoctoral researcher for a short period?), and know what you need to do in order to finish on time (Rule 1).

## Rule 9: Network

Unofficial statistics tell us that only around 30% of jobs are advertised, so to enhance your employment prospects you would be well advised to network in order to access the hidden job market. During the final year of your PhD, and even earlier, you can build up and extend your network so that your chances of finding the job of your choice are optimized. If you are looking for research positions, your supervisor might have contacts or know about positions available in academia or industry. Reviewing your personal network further will reveal it consists of colleagues, friends, and family. You may also have a wider network of collaborators (research and industry), people associated with your research whom you have met during the course of your PhD, as well as many others. Conferences, seminars, informal gatherings, and learned societies are great places to meet the academic community face to face or to broaden your horizons. Job fairs are held at universities and sometimes during conferences, where experts from industry look for potential employees as well as sometimes provide informal advice on your curriculum vitae (CV). Try to exploit these opportunities if they come your way.

A relatively recent, and highly democratic, addition to the networking system is social media, through which it is possible to meet people online from all over the world and from all walks of life. More and more professors, researchers, students, policy makers, science “celebrities”, science communicators, industry personnel, and professionals have a presence on social media, using it primarily for work-related purposes. Researchgate, LinkedIn, and Twitter are probably the most useful platforms for networking with academia, business, and the wider world, respectively. Your online profile should be fully completed and reflect your expertise, achievements, and personality. Used to greatest effect, social media will give you access to information, jobs, and influential people—its importance to you as a PhD student cannot be overestimated.

## Rule 10: Leave on Good Terms

Wrap up the work in your lab, especially if you are leaving the institute. This includes any required training of new personnel in the methods and techniques you use, having lab notes in order, making it easy for other lab members to access your protocols and data, organizing and labelling your reagents and equipment, and documenting your computer code. If someone is taking over an unfinished project from you, take time to hand it over. Discuss with your supervisor to find a solution for who will do the final experiments, how to proceed with the writing of journal manuscripts, and what should be the order of authorship. If you have started a project that you want to take with you to your new lab, discuss with your supervisor how to handle possible future publications and how to agree on material transfer. If your work resulted in patents or patentable innovations, make sure you are clear about regulations concerning patents and intellectual property, both at your PhD institution and at the institution to which you are moving. Stay in touch with your former colleagues and cultivate the contacts you have made in graduate school; they are sure to be useful during the course of your career.
